# Tolerogenic dendritic cells attenuate collagen-induced arthritis through a mucosal immunity–gut microbiota–butyrate axis

**DOI:** 10.3389/fimmu.2026.1971542

**Published:** 2026-10-29

**Authors:** Xiufang Wan, Yan Zhou, Tiaoyu Long, Rui Yuan, Lunmin Bao, Honghong Li, Ping Shi, Rui Gong, Shirong Yan, Qingqing Wu, Hongmei Jiang

**Affiliations:** 1 School of Clinical Laboratory Science, Guizhou Medical University, Guiyang, China; 2 Center for Clinical Laboratories, The Affiliated Hospital of Guizhou Medical University, Guiyang, China; 3 Department of Laboratory Medicine, The Second Affiliated Hospital of Guizhou University of Traditional Chinese Medicine, Guiyang, China; 4 Department of Laboratory Medicine, Anshun City People’s Hospital, Anshun, China; 5 Department of Tuberculosis, Affiliated Hospital of Zunyi Medical University, Zunyi, China; 6 Department of Medical Technology, Zunyi Medical and Pharmaceutical College, Zunyi, China

**Keywords:** butyrate, collagen-induced arthritis, fecal microbiota transplantation, gut microbiota, gut-joint axis, mucosal immunity, rheumatoid arthritis, tolerogenic dendritic cells

## Abstract

**Background:**

Tolerogenic dendritic cells (TolDCs) are being developed to restore antigen-specific immune tolerance in autoimmune diseases. However, whether their therapeutic effects in autoimmune arthritis involve modulation of the gut–joint axis remains unclear.

**Methods:**

TolDCs generated using an NF-κB decoy oligodeoxynucleotide were loaded with bovine type II collagen and administered to rats with collagen-induced arthritis (CIA). Arthritis severity was assessed clinically and histologically. Antibiotic-mediated microbiota depletion and fecal microbiota transplantation (FMT) were used to assess microbiota involvement. Colonic tight-junction protein expression, mesenteric lymph node Treg/Th17 balance, fecal microbial communities, PICRUSt2-predicted short-chain fatty acid (SCFA)-related metabolic functions based on 16S rRNA sequencing data, serum SCFAs, and the effects of sodium butyrate supplementation were also evaluated.

**Results:**

TolDCs reduced paw swelling and arthritis scores and ameliorated ankle-joint pathology. In the concurrent antibiotic-depletion experiment, TolDC-associated improvements in arthritis outcomes did not reach statistical significance, whereas FMT from TolDC-treated donors partially transferred protection to antibiotic-pretreated CIA recipients. TolDC treatment increased colonic ZO-1 and occludin expression, shifted the mesenteric lymph node Treg/Th17 balance toward a regulatory profile, altered gut microbial community structure and composition, and was accompanied by higher predicted abundances of selected butyrate-associated MetaCyc pathways and KEGG Orthology (KO) functional groups, along with increased serum butyrate concentrations. Sodium butyrate supplementation partially reproduced the protective phenotype.

**Conclusion:**

These findings support a microbiota-dependent component of TolDC-mediated protection and suggest that a coordinated mucosal immunity–gut microbiota–butyrate axis may contribute to arthritis control.

## 1 Introduction

Rheumatoid arthritis (RA) is a chronic autoimmune disease characterized by persistent synovitis, systemic immune dysregulation, and progressive cartilage and bone destruction (1), leading to pain, disability, and reduced quality of life (2, 3). Although advances in glucocorticoid and disease-modifying antirheumatic drug therapy have substantially improved disease control, including the use of conventional synthetic, biologic, and targeted synthetic agents, a substantial subset of patients remain refractory to treatment or experience adverse effects that necessitate treatment modification or discontinuation (4–6). Strategies that restore immune tolerance rather than broadly suppress immunity therefore remain an important therapeutic goal.

Tolerogenic dendritic cells (TolDCs) have attracted interest as a cellular immunotherapy because they can limit effector T-cell activation and promote regulatory immune responses (7). TolDCs typically display an immature or semimature phenotype with reduced expression of major histocompatibility complex class II and costimulatory molecules such as CD40, CD80, and CD86 (8). Because NF-κB signaling is a key regulator of dendritic-cell maturation and T-cell priming, NF-κB decoy oligodeoxynucleotides (ODNs) have been used to generate DCs with an immature and tolerogenic phenotype (9, 10). Our previous work showed that NF-κB ODN decoy-induced TolDCs attenuated CIA, but the mechanisms extending beyond direct systemic immunoregulation remained unclear (11, 12).

RA is increasingly recognized as a systemic autoimmune disorder whose initiation and progression are closely associated with mucosal immune dysfunction and gut microbial dysbiosis (13, 14). The gut–joint axis proposes that impaired epithelial barrier integrity, altered microbial communities, and dysregulated gut-associated immune responses can promote systemic inflammatory priming and joint disease (15, 16). Mesenteric lymph nodes (MLNs) are major sites where intestinal antigen-bearing dendritic cells convey microbial signals to prime and polarize T cells. Accordingly, disruption of the Th17/Treg balance in MLNs may provide a mechanistic link between mucosal immune dysregulation and autoimmune arthritis (17, 18).

Microbial metabolites provide an additional mechanistic link between intestinal ecology and host immunity. Short-chain fatty acids (SCFAs), particularly butyrate, regulate epithelial barrier integrity, T-cell differentiation, macrophage function, cytokine production, and osteoimmune responses through G protein-coupled receptor signaling and histone deacetylase inhibition (19, 20). Altered abundance of SCFA-associated bacteria and reduced butyrate availability have been implicated in RA and experimental arthritis (21, 22). Whether tolerance-inducing cellular therapies can reshape these microbial metabolic signals is not well understood.

Dendritic cells are strategically positioned to translate microbial cues into local and systemic immune responses. Arthritis-associated gut microbes can prime dendritic cells to promote pathogenic Th17 differentiation, whereas enhanced tolerogenic dendritic-cell activity has been shown to reshape gut microbial communities in metabolic disease (23, 24). We previously observed that adoptively transferred TolDCs migrate to and accumulate in the MLNs of CIA rats, where they modulate immune-checkpoint expression and reduce arthritis severity (11). These findings raised the possibility that TolDCs influence arthritis by restoring the intestinal immune microenvironment and, in turn, reshaping microbiota-derived metabolic signals.

The present study tested whether NF-κB ODN decoy-induced TolDCs alleviate CIA through a mucosal immunity–gut microbiota–butyrate axis. We characterized the TolDC product, evaluated its therapeutic effects, tested microbiota involvement using antibiotic depletion and FMT, assessed intestinal barrier integrity and MLN Treg/Th17 balance, profiled gut microbial communities and serum SCFAs, and examined whether butyrate supplementation could reproduce part of the protective phenotype. This integrated design was intended to move beyond compositional association and provide functional evidence for a TolDC-regulated gut–joint pathway.

## 2 Materials and methods

### 2.1 Animals, housing, and study design

Female Sprague–Dawley (SD) rats weighing 120–140 g were obtained from Beijing Vital River Laboratory Animal Technology Co., Ltd. (Beijing, China). Rats were maintained under specific pathogen-free conditions at 22 ± 2 °C and 55 ± 5% relative humidity on a 12-h light/dark cycle, with food and water available ad libitum. After 7 days of acclimatization, rats designated for CIA induction underwent the immunization protocol described below. Rats meeting the prespecified criterion for successful CIA induction were randomly allocated to the CIA, TolDC, antibiotic cocktail (ABX), and TolDC + ABX groups, while non-immunized rats served as Control animals. These groups were established concurrently and evaluated on day 49; separate recipient cohorts were used for the FMT and Butyrate experiments. All procedures were approved by the Animal Ethics Committee of Guizhou Medical University (Approval No. 2304005) and complied with institutional requirements.

### 2.2 Isolation and culture of tolerogenic dendritic cells

Donor SD rats were deeply anesthetized with 3–5% isoflurane (RWD Life Science Co., Ltd., Shenzhen, China; Cat. No. R510-22-10) and euthanized by exsanguination under anesthesia. After death was confirmed, both tibiae were aseptically removed and flushed with sterile phosphate-buffered saline (PBS). The marrow suspension was passed through a sterile 200-mesh strainer, and erythrocytes were removed with red blood cell lysis buffer (Solarbio, Beijing, China; Cat. No. R1010).

After centrifugation at 500 × g for 5 min, cells were resuspended at 1 × 10^6^ cells/mL in RPMI 1640 (Gibco, Grand Island, NY, USA; Cat. No. C11875500BT) supplemented with 10% fetal bovine serum (ScienCell Research Laboratories, Carlsbad, CA, USA; Cat. No. 0500) and 1% penicillin–streptomycin (Gibco; Cat. No. 15140122). Cells were allowed to adhere for 90 min at 37 °C and 5% CO_2_; nonadherent cells were removed, and the adherent fraction was cultured with recombinant rat interleukin-4 (20 ng/mL; PeproTech, Cranbury, NJ, USA; Cat. No. 400-04-20UG) and granulocyte–macrophage colony-stimulating factor (30 ng/mL; PeproTech; Cat. No. 400-23-20UG). The seeding day was designated day 0, and half of the medium was replaced on days 3 and 5 with fresh cytokine-supplemented medium.

To generate TolDCs, a fully phosphorothioate-modified NF-κB decoy ODN was added on day 0 at 10 μM. The sense and antisense strands (5′-AGGGACTTTCCGCTGGGGACTTTCC-3′ and 5′-GGAAAGTCCCCAGCGGAAAGTCCCT-3′, respectively) were synthesized and purified by ULTRAPAGE (Sangon Biotech, Shanghai, China). The sense strand contained two copies of the NF-κB-binding motif GGGACTTTCC. Equimolar strands were mixed in 150 mM NaCl, heated to 100 °C, and cooled to room temperature to form the double-stranded decoy.

On day 5, cells were pulsed for 24 h with immunization-grade bovine type II collagen (CII; 50 μg/mL; Chondrex, Woodinville, WA, USA; Cat. No. 20021) and harvested on day 6 for characterization or adoptive transfer.

### 2.3 Morphological and phenotypic characterization of TolDCs

Cell morphology was assessed on day 6 by Giemsa staining. Cells were washed twice with PBS, air-dried on glass slides, fixed in methanol for 3 min, and stained with Giemsa solution (Solarbio; Cat. No. G1010). Images were acquired with a BX53 light microscope (Olympus Corporation, Tokyo, Japan) at 1,000× total magnification. Three independent preparations were examined, with 10 randomly selected fields per preparation.

For flow cytometry, 1 × 10^5^ cells per tube were stained with Alexa Fluor 647 anti-rat OX-62/CD103 (2 μL/test; clone OX-62; BioLegend, San Diego, CA, USA; Cat. No. 205509), PE anti-rat CD80 (1.5 μL/test; clone 3H5; BioLegend; Cat. No. 200205), and FITC anti-rat CD86 (2 μL/test; clone 24F; BioLegend; Cat. No. 200305; RRID: AB_313854).

Cells were incubated for 30 min at room temperature in the dark, washed twice, and resuspended in 400 μL staining buffer. At least 30,000 events per sample were acquired on a Navios flow cytometer (Beckman Coulter, Brea, CA, USA). Compensation was established with single-stained cells, and unstained cells were used to assess background fluorescence and set gates.

Data were analyzed in FlowJo version 10.0.7 (BD Life Sciences, Ashland, OR, USA; RRID: SCR_008520). The main cell population was selected by forward- and side-scatter area, after which OX-62 positivity and CD80/CD86 expression were quantified. Three independent cell preparations were analyzed.

### 2.4 CIA induction and experimental interventions

#### 2.4.1 CIA induction and TolDC administration

CIA was induced with immunization-grade bovine CII according to a published protocol (11), with minor modifications. On day 0, each rat received 200 μg CII emulsified 1:1 with complete Freund’s adjuvant (Sigma-Aldrich, St. Louis, MO, USA; Cat. No. F5881) in a total volume of 200 μL by a single subcutaneous injection into the right hind paw pad. On day 14, rats received 200 μg CII emulsified 1:1 with incomplete Freund’s adjuvant (Sigma-Aldrich; Cat. No. F5506) in 200 μL by subcutaneous injection at the base of the tail. Before treatment allocation on day 21, arthritis severity was assessed as described in Section 2.5, and rats with a total arthritis score ≥4 were considered to have successfully established CIA and were eligible for treatment-group allocation (25). On day 21, rats assigned to the TolDC and TolDC + ABX groups received 5 × 10^6^ CII-loaded TolDCs in 500 μL PBS by tail-vein injection. Corresponding vehicle-treated groups received 500 μL PBS.

#### 2.4.2 Antibiotic-mediated microbiota depletion and fecal microbiota transplantation

In the concurrent primary cohort, rats in the ABX and TolDC + ABX groups received ampicillin (0.5 g/L; Solarbio, Beijing, China; Cat. No. A8180), streptomycin (1 g/L; Cat. No. S8290), vancomycin (0.5 g/L; Cat. No. V8050), and gentamicin (1 g/L; Cat. No. G8171) in drinking water from day 21 through day 49. The solution was prepared fresh and replaced every 2 days. Thus, the CIA versus TolDC comparison assessed TolDC efficacy without antibiotics, whereas the ABX versus TolDC + ABX comparison assessed efficacy during microbiota depletion.

For FMT, recipient CIA rats received the same antibiotic cocktail from day 21 to day 28; antibiotics were discontinued before the first transplant. Feces were collected on days 42–49 from untreated CIA donors or CIA donors treated with CII-loaded TolDCs. Samples from 8 donors per group were pooled in equal amounts and stored at −80 °C. Before each administration, pooled feces were thawed, suspended in sterile PBS at 100 mg/mL, homogenized, and centrifuged at 500 × g for 3 min at room temperature. The supernatant was administered by oral gavage at 10 mL/kg, equivalent to 1 g starting fecal material/kg. Beginning on day 28, recipients received FMT once daily for 28 consecutive days and were designated CIA-FMT or TolDC-FMT according to donor source. Fecal samples collected on day 28 were used to verify antibiotic-associated loss of microbial richness and diversity; endpoint samples were collected on day 56 to assess recovery after FMT.

#### 2.4.3 Sodium butyrate supplementation

Rats in the Butyrate group received sodium butyrate (150 mM; Solarbio, Beijing, China; Cat. No. IS0190; purity ≥98%) ad libitum in drinking water from day 21 to day 49. Fresh sodium butyrate solution was prepared and replaced every 2 days. Rats in the CIA and Control groups had ad libitum access to regular drinking water. At the endpoint on day 49, rats were anesthetized with isoflurane using an R500 small animal anesthesia machine (RWD Life Science Co., Ltd., Shenzhen, China). Anesthesia was induced with 3–5% isoflurane and maintained with 1–3% isoflurane. Adequate anesthetic depth was confirmed by the absence of the pedal withdrawal reflex. Under deep anesthesia, blood was collected from the abdominal aorta as a terminal procedure, and the animals were euthanized by exsanguination. Death was confirmed by the absence of spontaneous respiration and heartbeat. Serum, fecal or cecal contents, colon, mesenteric lymph nodes (MLNs), ankle joints, and paw tissues were collected as required for the analyses described below.

### 2.5 Assessment of arthritis severity

Arthritis was evaluated weekly using a 0–4 score for each paw: 0, no arthritis; 1, swelling of one or two digits; 2, swelling of more than two digits or mild whole-paw swelling; 3, pronounced swelling of the whole paw and ankle; and 4, severe swelling, deformity, or ankylosis (26). Scores from all four paws were summed to a maximum of 16. The maximum mediolateral width of each hind ankle was measured in millimeters with a digital caliper (Mitutoyo Corporation, Kawasaki, Kanagawa, Japan; Model No. 500-196-30). Three investigators blinded to group allocation independently performed scoring and measurements. Scores were averaged across investigators; hind-ankle width measurements were averaged first across investigators for each hind ankle and then across both hind ankles to obtain one value per rat.

### 2.6 Histopathological evaluation

Ankle joints were fixed in 4% paraformaldehyde for 24 h and decalcified in 12.5% EDTA disodium salt (pH 7.4) for 30–35 days, with solution replacement every other day. Decalcification was considered complete when the tissue was soft, rubber-like, and readily penetrated with a needle. Specimens were bisected sagittally, paraffin-embedded, sectioned at 5 μm, and stained with hematoxylin and eosin. Synovial hyperplasia was scored from 0 to 3 (normal to severe with pannus), and inflammatory-cell infiltration from 0 to 3 (none to severe), using a prespecified semiquantitative scale. The two components were summed to a 0–6 composite score. Ten randomly selected fields per section were examined at 400× with a BX53 microscope. Two investigators blinded to group allocation scored each specimen independently, and their mean was used for analysis.

### 2.7 Immunohistochemical analysis of intestinal tight-junction proteins

Colon samples were fixed in 4% paraformaldehyde for 24 h, paraffin-embedded, and sectioned at 5 μm. After deparaffinization and rehydration, antigen retrieval was performed in EDTA buffer (pH 9.0) at 95 °C for 20 min. Endogenous peroxidase was blocked with 3% hydrogen peroxide for 15 min, followed by 5% bovine serum albumin for 30 min. Separate sections were incubated overnight at 4 °C with rabbit polyclonal anti-ZO-1 (1:400; Proteintech, Wuhan, China; Cat. No. 21773-1-AP; RRID: AB_10733242) or anti-occludin (1:400; Proteintech; Cat. No. 27260-1-AP; RRID: AB_2880820). After three washes with Tris-buffered saline containing 0.1% Tween-20, sections were incubated for 60 min at room temperature with a rabbit-specific horseradish peroxidase polymer reagent (ZSGB-BIO, Beijing, China; Cat. No. PV-6001), developed with 3,3′-diaminobenzidine (Cat. No. PV-6000D) for 1–2 min, counterstained with hematoxylin, dehydrated, cleared, and mounted. Whole-slide images were acquired on an Aperio VERSA scanner (Leica Biosystems, Vista, CA, USA). For each marker, one section per animal and 10 randomly selected, non-overlapping mucosal fields at 400× were analyzed with ImageJ version 1.53t (National Institutes of Health, Bethesda, MD, USA; RRID: SCR_003070). A threshold established from negative controls was applied uniformly, and expression was quantified as mean optical density. Field-level measurements were averaged to one value per animal, and results from two blinded investigators were averaged.

### 2.8 Flow-cytometric analysis of Treg and Th17 cells in MLNs

MLNs were mechanically dissociated through a 70-μm strainer, centrifuged at 500 × g for 5 min, and resuspended in RPMI 1640 containing 10% fetal bovine serum. Viability was assessed by trypan blue exclusion, and 1 × 10^6^ cells in 200 μL were stimulated for 5 h at 37 °C and 5% CO_2_ with Cell Activation Cocktail with Brefeldin A (2 μL/mL; BioLegend; Cat. No. 423304), yielding final concentrations of 81 nM phorbol 12-myristate 13-acetate, 1.34 μM ionomycin, and 5 μg/mL brefeldin A. Cells were stained with Zombie Aqua Fixable Viability Dye (1:1,000; BioLegend; Cat. No. 423101), FITC anti-rat CD4 (2 μL/test; clone OX-35; Cat. No. 203305; RRID: AB_1501180), and PE anti-rat CD25 (1.25 μL/test; clone OX-39; Cat. No. 202105; RRID: AB_314002). After fixation and permeabilization with the BD Pharmingen Transcription Factor Buffer Set (BD Biosciences, San Jose, CA, USA; Cat. No. 562574), cells were stained with eFluor 660 anti-Foxp3 (2.5 μL/test; clone FJK-16s; Thermo Fisher Scientific; Cat. No. 50-5773-82; RRID: AB_11218868) and PE-Cyanine7 anti-IL-17A (2.5 μL/test; clone eBio17B7; Cat. No. 25-7177-82; RRID: AB_10732356). At least 30,000 events per sample were acquired on a Navios cytometer. Single-stained controls were used for compensation, unstained cells for background, and fluorescence-minus-one controls for CD25, Foxp3, and IL-17A gates. In FlowJo version 10.0.7, lymphocytes were selected by FSC-A/SSC-A, followed by singlets, viable cells, and CD4^+^ T cells. Tregs were defined as CD4^+^CD25^+^Foxp3^+^ cells and Th17 cells as CD4^+^IL-17A^+^ cells; both frequencies were calculated among viable singlet CD4^+^ T cells.

### 2.9 16S rRNA gene sequencing and microbiota analysis

Fecal DNA was extracted using a cetyltrimethylammonium bromide method. Samples were placed in 2-mL tubes with two sterile steel beads, snap-frozen in liquid nitrogen, mechanically homogenized, and incubated for 1 h at 65 °C with 600 μL cetyltrimethylammonium bromide solution and 12 μL β-mercaptoethanol, with inversion every 15 min. After extraction with an equal volume of chloroform and centrifugation at 12,000 × g for 5 min, the aqueous phase was precipitated with two volumes of ethanol prechilled to −20 °C. Pellets were recovered at 12,000 × g for 5 min, dissolved in 20 μL double-distilled water, and assessed by 1% agarose electrophoresis and microvolume spectrophotometry.

The bacterial 16S rRNA V3–V4 region was amplified with primers 338F (5′-ACTCCTACGGGAGGCAGCAG-3′) and 806R (5′-GGACTACHVGGGTWTCTAAT-3′): 95 °C for 3 min; 33 cycles of 95 °C for 30 s, 54 °C for 30 s, and 72 °C for 45 s; and 72 °C for 10 min. Purified amplicons were pooled equimolarly and sequenced as 2 × 250-bp reads on an Illumina NovaSeq platform (Majorbio Bio-Pharm Technology Co., Ltd., Shanghai, China). Reads were demultiplexed, quality-filtered, and merged; amplicon sequence variants were inferred with DADA2 in QIIME 2 version 2020.2 and classified against SILVA release 138. Chimeric, mitochondrial, chloroplast, nonbacterial, and unassigned bacterial-kingdom sequences were removed, and samples were rarefied to 20,000 reads. Shannon and Chao1 indices were calculated at the amplicon sequence variant level. Bray–Curtis dissimilarities were visualized by principal coordinates analysis (PCoA), and overall differences in community structure among groups were evaluated by analysis of similarities (ANOSIM) with 999 permutations. Genus-level core curves, UpSet plots, and hierarchical clustering were also generated. Exploratory functional prediction of the 16S rRNA amplicon sequence variants was performed using PICRUSt2 version v2.2.0. Differential taxa were assessed using Kruskal–Wallis tests, with false-discovery-rate correction across taxa. Taxa with significant overall differences were further evaluated using Tukey–Kramer *post-hoc* tests, and pairwise results were used for direction-specific between-group interpretation. Eight rats per group were analyzed. Sequencing data are deposited under BioProject PRJNA1110009.

### 2.10 Measurement of serum short-chain fatty acids

Serum SCFAs were quantified by liquid chromatography–electrospray ionization–tandem mass spectrometry after 3-nitrophenylhydrazine derivatization (Majorbio Bio-Pharm Technology Co., Ltd.). Serum (50 μL) was mixed with 100 μL acetonitrile containing internal standards, sonicated at 5 °C for 30 min, and centrifuged at 13,000 × g for 15 min at 4 °C. The supernatant was reacted for 30 min at 40 °C with 20 μL 200 mM 3-nitrophenylhydrazine hydrochloride and 20 μL 120 mM 1-ethyl-3-(3-dimethylaminopropyl)carbodiimide hydrochloride containing 6% pyridine, then brought to 750 μL with 50% acetonitrile. Separation was performed on an ExionLC AD system with an ACQUITY UPLC BEH C18 column (150 × 2.1 mm, 1.7 μm; 40 °C) coupled to a QTRAP 6500+ mass spectrometer (SCIEX). Mobile phases were water and acetonitrile, each containing 0.1% formic acid. The gradient was 10% B at 0–2 min, 10–55% B at 2–11 min, 55–95% B at 11–12 min, 95% B at 12–13 min, and return to 10% B by 16 min. Negative-ion multiple-reaction monitoring used curtain gas 35 psi, collision gas medium, ion-spray voltage −4,500 V, source temperature 450 °C, and ion-source gases 1 and 2 at 40 psi. Weighted 1/x calibration ranges were 0.5–2,000 ng/mL for acetate; 0.2–200 ng/mL for propionate, isobutyrate, and butyrate; 0.1–100 ng/mL for isovalerate and valerate; 0.05–20 ng/mL for 4-methylvalerate; and 0.1–200 ng/mL for caproate. Coefficients of determination were 0.9927–0.9966. Analyte-to-internal-standard peak-area ratios were quantified in SCIEX OS and reported as ng/mL. Hemolyzed serum specimens were excluded from targeted SCFA quantification because they did not meet sample-quality requirements. Consequently, five non-hemolyzed serum samples per group were available for SCFA analysis (n = 5 per group).

### 2.11 Statistical analysis

Analyses were performed in GraphPad Prism version 9.4.1 (GraphPad Software, San Diego, CA, USA). Normality was assessed by the Shapiro–Wilk test. Normally distributed data are presented as mean ± SEM and were analyzed by two-tailed unpaired Student’s t-test or one-way ANOVA with Tukey’s multiple-comparisons test, as appropriate. Non-normally distributed data are presented as median and interquartile range and were analyzed by Mann–Whitney U or Kruskal–Wallis tests with Dunn’s multiple-comparisons test. Longitudinal hind-ankle width and arthritis-score data were analyzed by two-way repeated-measures ANOVA with group and time as factors; the group × time interaction was included, Geisser–Greenhouse correction was applied when required, and prespecified comparisons at individual time points used Šídák adjustment. For exploratory PICRUSt2 MetaCyc pathway and KO comparisons, group differences were assessed using the Kruskal–Wallis test followed by Dunn’s *post hoc* multiple-comparisons test. For beta-diversity analysis, between-group differences in Bray–Curtis dissimilarity were evaluated by ANOSIM with 999 permutations. Differential-abundance omnibus P values were adjusted for false discovery rate, and Tukey–Kramer *post-hoc* tests were used for pairwise interpretation of taxa with significant overall differences. Spearman correlations were calculated from paired microbiome and serum metabolomic data from the same 15 rats, and the correlation P values were adjusted for multiple testing. Associations that remained significant after adjustment were reported but interpreted as exploratory because of the modest sample size and correlational design. Technical replicates were averaged to one biological value per animal. All tests were two-sided; P < 0.05 or the prespecified adjusted significance threshold was considered statistically significant, as applicable.

## 3 Results

### 3.1 NF-κB decoy ODN treatment generates OX-62^+^ dendritic cells with an immature phenotype

Bone marrow-derived cells were differentiated in the absence or presence of NF-κB decoy ODN to generate conventional dendritic cells and TolDCs, respectively. On culture day 5, both preparations were pulsed with bovine type II collagen (CII; 50 μg/mL) for 24 h and collected on day 6 (**Figure 1A**). More than 97% of cells in both groups were OX-62^+^, with no between-group difference, whereas the frequencies of CD80^+^ and CD86^+^ cells were lower in TolDC cultures (both P < 0.001; **Figures 1B, C**). Thus, NF-κB decoy ODN treatment generated an OX-62^+^ population with reduced costimulatory-molecule expression and an immature phenotype.

**Figure 1:**
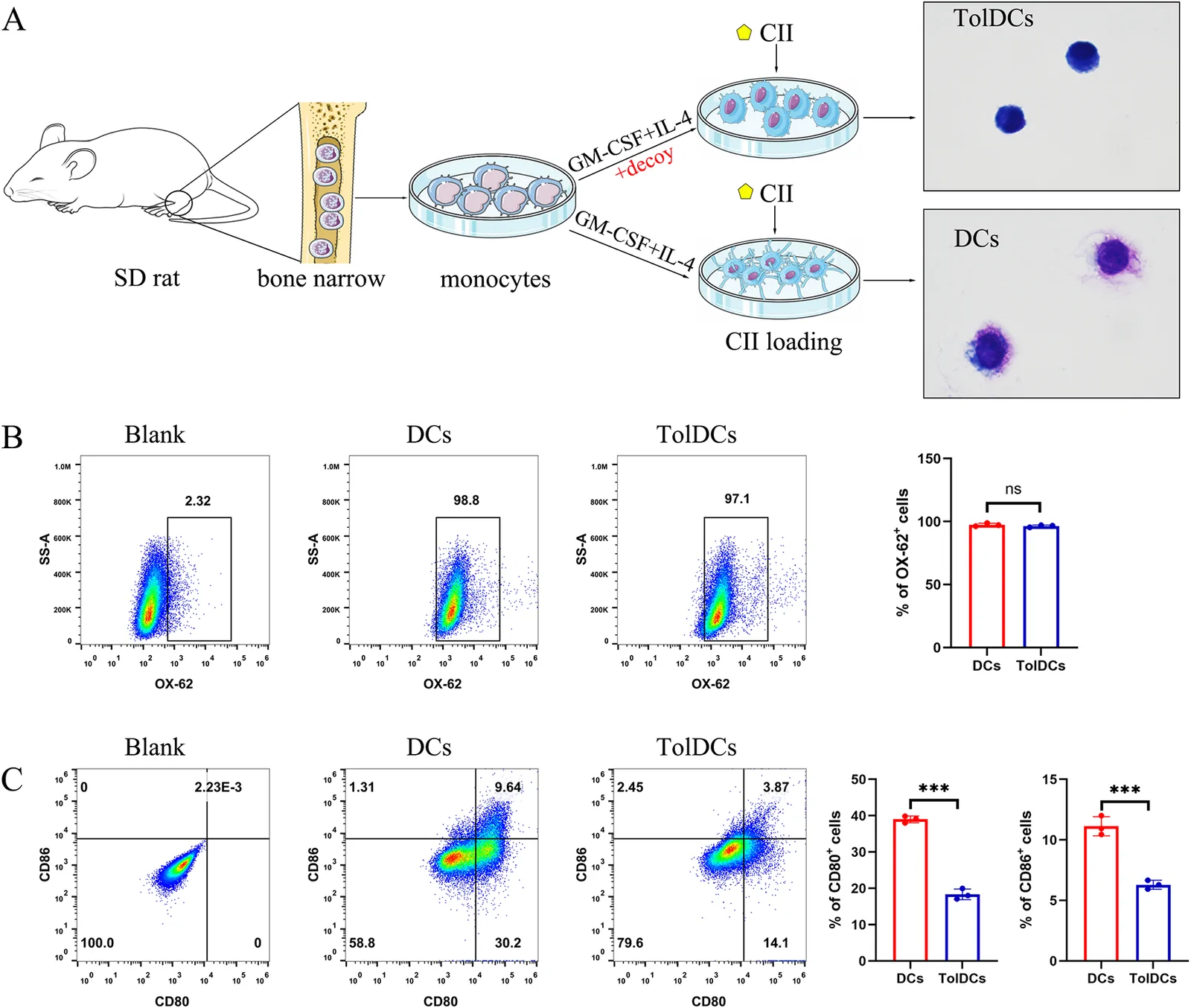
Generation and phenotypic characterization of CII-loaded NF-κB decoy ODN-induced tolerogenic dendritic cells. **(A)** Culture and CII-loading workflow and representative Giemsa-stained conventional dendritic cells (DCs) and tolerogenic dendritic cells (TolDCs). Images are representative of three independent preparations. **(B)** Representative flow-cytometry plots and frequency of OX-62^+^ cells. Blank denotes unstained cells used to establish background fluorescence and gating thresholds. **(C)** Representative plots and frequencies of CD80^+^ and CD86^+^ cells. Quantitative data are mean ± SEM; n = 3 independent preparations. Comparisons used two-tailed unpaired Student’s t-tests. ns, P > 0.05; ***P < 0.001. DC, dendritic cell; ODN, oligodeoxynucleotide; TolDC, tolerogenic dendritic cell; CII, type II collagen.

### 3.2 TolDC treatment attenuates clinical arthritis and joint pathology

Following CII immunization, CIA rats developed progressive paw swelling and increasing arthritis scores. On day 49, right hind-paw swelling was less pronounced in the TolDC group than in the CIA group (**Figure 2B**). From day 28 onward, mean hind-ankle width and total arthritis scores were lower in TolDC-treated rats at the indicated time points (**Figures 2C, D**). Histologically, CIA joints showed synovial hyperplasia, inflammatory-cell infiltration, and disruption of cartilage and subchondral bone architecture, whereas TolDC-treated joints showed less inflammation and greater structural preservation (**Figure 2E**). Composite histopathological scores were likewise lower after TolDC treatment (**Figure 2F**). These findings establish that TolDC administration attenuated clinical and histological arthritis in the absence of antibiotic treatment.

**Figure 2:**
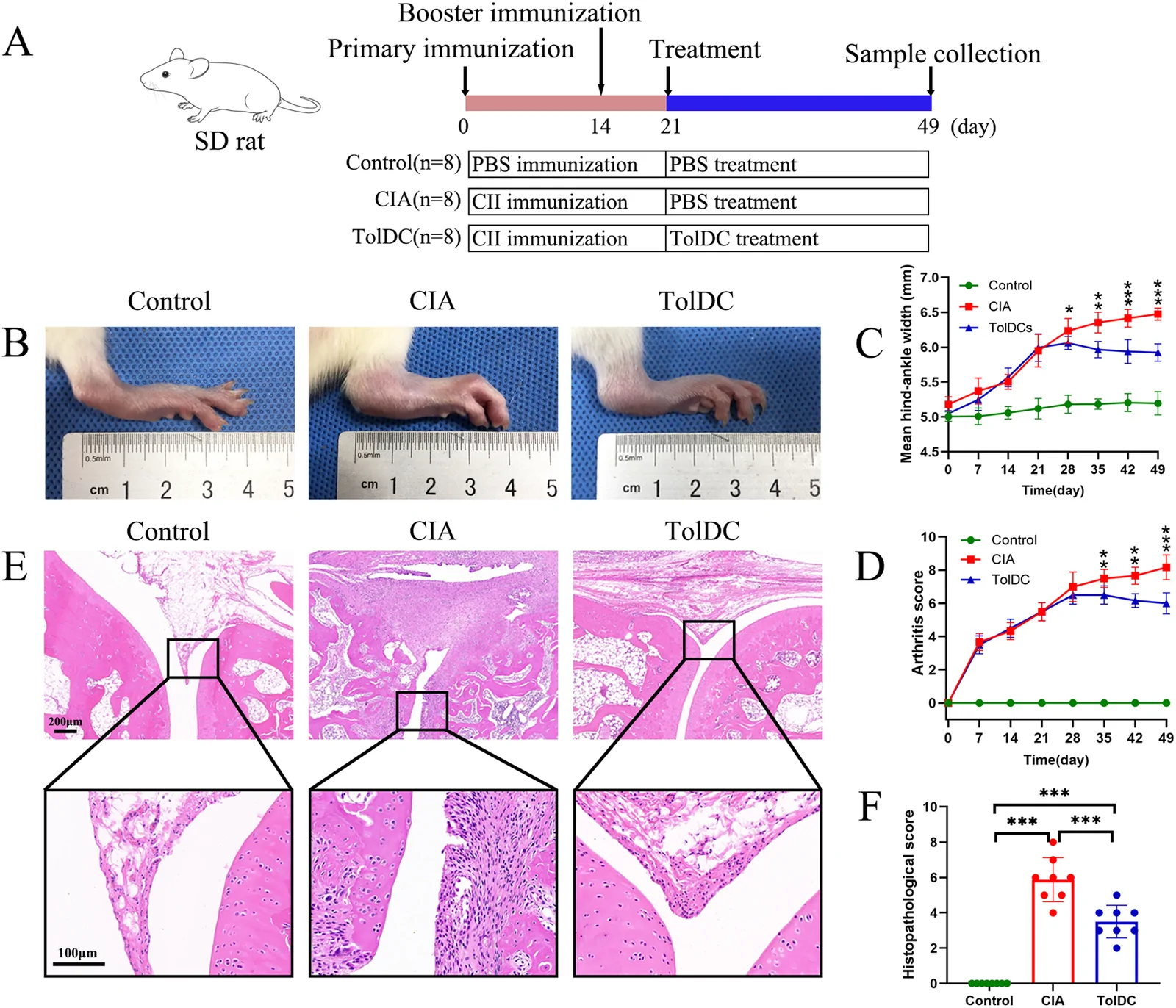
TolDC administration attenuates clinical arthritis and ankle-joint histopathology in CIA rats. **(A)** Experimental timeline. Rats received primary and booster immunization with bovine type II collagen (CII) on days 0 and 14, respectively. On day 21, rats received CII-loaded TolDCs or PBS; samples were collected on day 49. **(B)** Representative right hind paws on day 49. **(C)** Mean width of both hind ankles measured weekly. **(D)** Total arthritis scores for all four paws (maximum, 16). **(E)** Representative hematoxylin and eosin-stained ankle joints. Scale bars, 200 μm in the upper panels and 100 μm in the enlarged lower panels. **(F)** Composite histopathological scores for synovial hyperplasia and inflammatory-cell infiltration (range, 0–6). Data are mean ± SEM; n = 8 biologically independent rats per group. Longitudinal data were analyzed by two-way repeated-measures ANOVA followed by Šídák’s comparisons. Asterisks in **(C)** and **(D)** denote CIA-versus-TolDC comparisons at the corresponding time points. Histopathological scores were analyzed by one-way ANOVA with Tukey’s test. *P < 0.05, **P < 0.01, ***P < 0.001. CIA, collagen-induced arthritis; CII, type II collagen; TolDC, tolerogenic dendritic cell.

### 3.3 Antibiotic administration attenuates TolDC-mediated amelioration of CIA

The CIA, TolDC, ABX, and TolDC + ABX groups were studied concurrently, allowing the therapeutic efficacy of TolDCs to be evaluated in the presence or absence of antibiotic treatment. Although TolDC administration significantly improved clinical and histopathological outcomes in CIA rats without antibiotic treatment (**Figure 2**), the TolDC + ABX group exhibited numerically lower paw swelling, clinical arthritis scores, and histopathological scores than the ABX group, but these differences did not reach statistical significance (**Figures 3B–F**), suggesting that antibiotic administration may attenuate the therapeutic effects of TolDCs in CIA rats. Furthermore, in a separate experimental cohort, antibiotic treatment significantly reduced gut microbial richness and diversity before FMT (**Figures 4C, D**), supporting the effectiveness of the antibiotic regimen in depleting the gut microbiota. Taken together, these findings suggest that antibiotic-mediated gut microbiota depletion may attenuate TolDC-mediated protection against CIA, supporting an important contributory role of the gut microbiota in the therapeutic effects of TolDCs.

**Figure 3:**
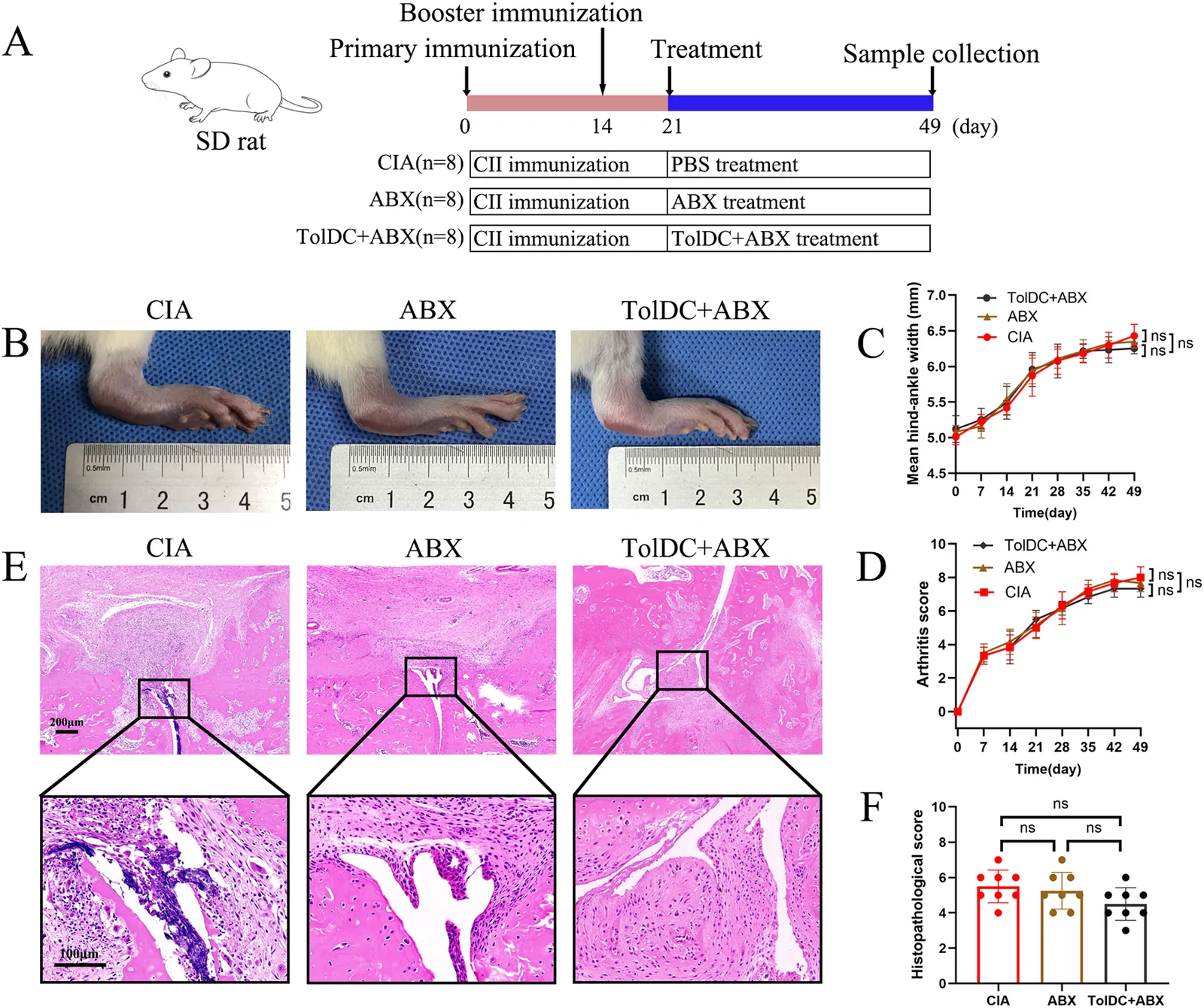
Antibiotic administration attenuates TolDC-mediated amelioration of CIA. **(A)** Experimental timeline. Rats received CII immunization on days 0 and 14. Beginning on day 21, they received PBS, an antibiotic cocktail (ABX), or TolDCs together with ABX; ABX treatment continued through day 49. The CIA, TolDC, ABX, and TolDC + ABX groups were studied concurrently, although the TolDC-only data are presented in **Figure 2**. **(B)** Representative right hind paws on day 49. **(C)** Mean width of both hind ankles measured weekly. **(D)** Total arthritis scores for all four paws (maximum, 16). **(E)** Representative hematoxylin and eosin-stained ankle joints. Scale bars, 200 μm in the upper panels and 100 μm in the enlarged lower panels. **(F)** Composite histopathological scores (range, 0–6). Data are mean ± SEM; n = 8 biologically independent rats per group. Longitudinal data were analyzed by two-way repeated-measures ANOVA, with Šídák’s comparisons among the displayed groups on day 49; histopathological scores were analyzed by one-way ANOVA with Tukey’s test. No significant differences were detected among the displayed groups. ns, P > 0.05. ABX, antibiotic cocktail; CIA, collagen-induced arthritis; CII, type II collagen; TolDC, tolerogenic dendritic cell.

**Figure 4:**
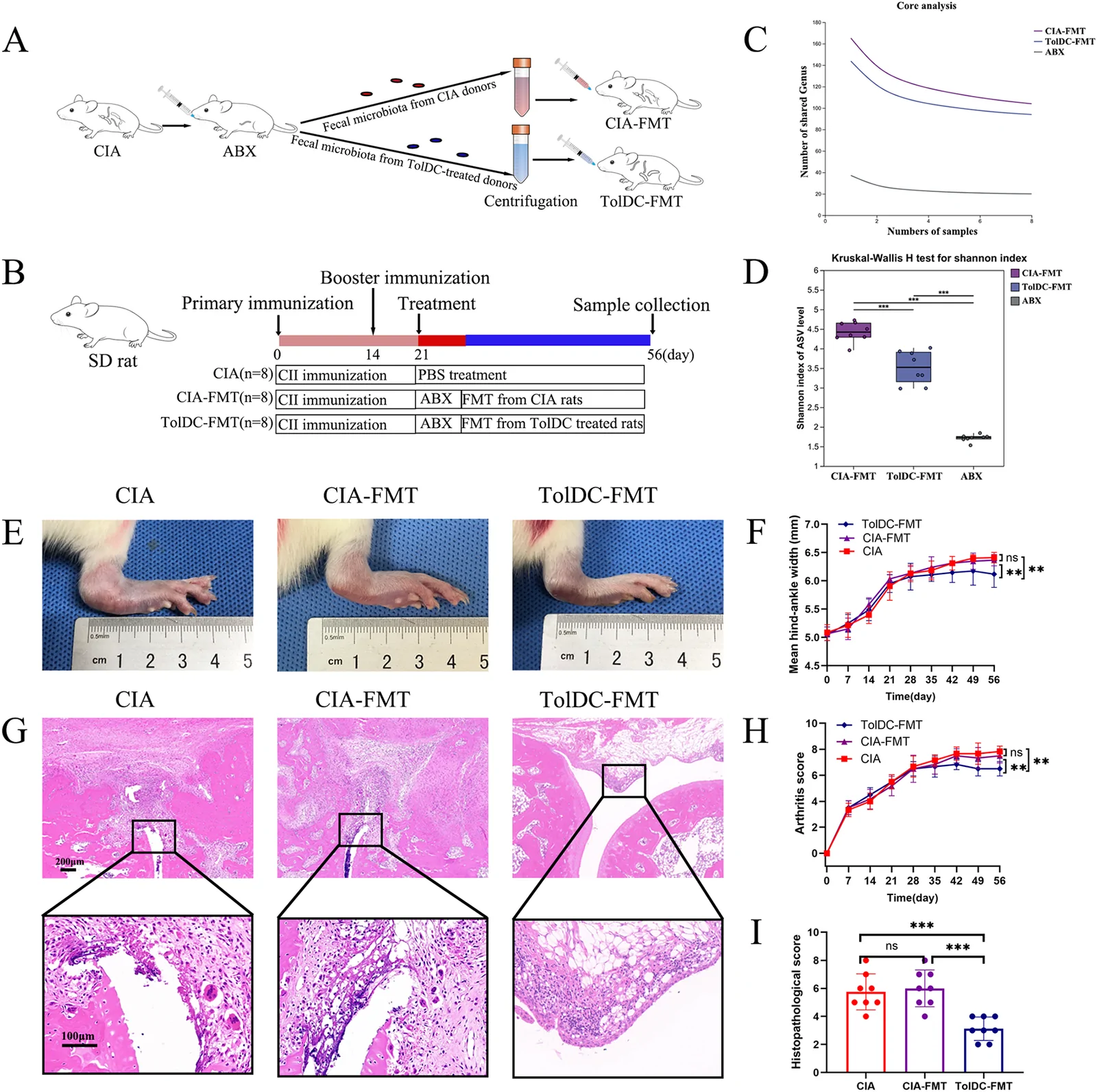
Fecal microbiota transplantation from TolDC-treated donors partially transfers the protective phenotype. **(A)** FMT procedure. Fecal suspensions from untreated CIA or TolDC-treated CIA donors were administered to antibiotic-pretreated CIA recipients, generating CIA-FMT and TolDC-FMT groups. **(B)** Experimental timeline. Recipients were immunized with CII on days 0 and 14, received ABX from day 21 to day 28, and underwent daily FMT for 28 consecutive days beginning on day 28; samples were collected on day 56. **(C)** Core genus curves showing genera shared across increasing numbers of samples after ABX pretreatment and after FMT. **(D)** Shannon diversity in post-ABX samples collected immediately before the first FMT and in endpoint CIA-FMT and TolDC-FMT samples. Post-ABX samples represent a pretreatment time point, not a separate treatment group. **(E)** Representative right hind paws on day 56. **(F)** Mean width of both hind ankles. **(G)** Representative hematoxylin and eosin-stained ankle joints. Scale bars, 200 μm in the upper panels and 100 μm in the enlarged lower panels. **(H)** Total arthritis scores (maximum, 16). **(I)** Composite histopathological scores (range, 0–6). Microbiota analyses used eight biologically independent rats per condition or time point (n = 8); Shannon diversity was analyzed by Kruskal–Wallis test with Dunn’s comparisons. Clinical and histopathological data are mean ± SEM from eight biologically independent rats per group (n = 8). Longitudinal outcomes were analyzed by two-way repeated-measures ANOVA with Šídák’s comparisons on day 56; histopathological scores were analyzed by one-way ANOVA with Tukey’s test. ns, P > 0.05; **P < 0.01; ***P < 0.001. ABX, antibiotic cocktail; CIA, collagen-induced arthritis; CII, type II collagen; FMT, fecal microbiota transplantation; TolDC, tolerogenic dendritic cell.

### 3.4 FMT from TolDC-treated donors partially transfers the protective phenotype

To test whether TolDC-associated microbial changes were transferable, antibiotic-pretreated CIA recipients received fecal microbiota from untreated CIA donors or TolDC-treated CIA donors, generating the CIA-FMT and TolDC-FMT groups (**Figures 4A, B**). Post-ABX samples contained fewer genera shared across animals and had lower Shannon diversity than endpoint samples from either FMT group, consistent with microbiota depletion followed by partial recovery after transplantation (**Figures 4C, D**). Shannon diversity remained higher in CIA-FMT than TolDC-FMT recipients, indicating that recolonization did not produce identical community states. These diversity measures support recovery but do not establish donor-strain engraftment. On day 56, CIA-FMT recipients did not improve relative to untreated CIA rats, whereas TolDC-FMT recipients showed less paw swelling, lower hind-ankle width and arthritis scores, and reduced joint pathology (**Figures 4E–I**). Thus, microbiota from TolDC-treated donors partially transferred the disease-ameliorating phenotype, providing functional transfer evidence that transferable microbial features contribute to TolDC-associated protection.

### 3.5 TolDC treatment increases colonic tight-junction proteins and shifts MLN T-cell balance

CIA rats showed lower colonic ZO-1 and occludin expression than Control rats, whereas both proteins were increased by TolDC treatment (all indicated P < 0.001; **Figures 5A–D**). These changes are consistent with partial recovery of a barrier-associated epithelial phenotype but do not directly measure intestinal permeability. In MLNs, CIA reduced the frequency of CD4^+^CD25^+^Foxp3^+^ Tregs, increased CD4^+^IL-17A^+^ Th17 cells, and lowered the Treg/Th17 ratio. TolDC treatment increased Tregs, reduced Th17 cells, and increased the ratio relative to CIA (**Figures 5F–J**). Together, these findings link TolDC treatment to coordinated changes in colonic tight-junction protein expression and mucosal T-cell balance.

**Figure 5:**
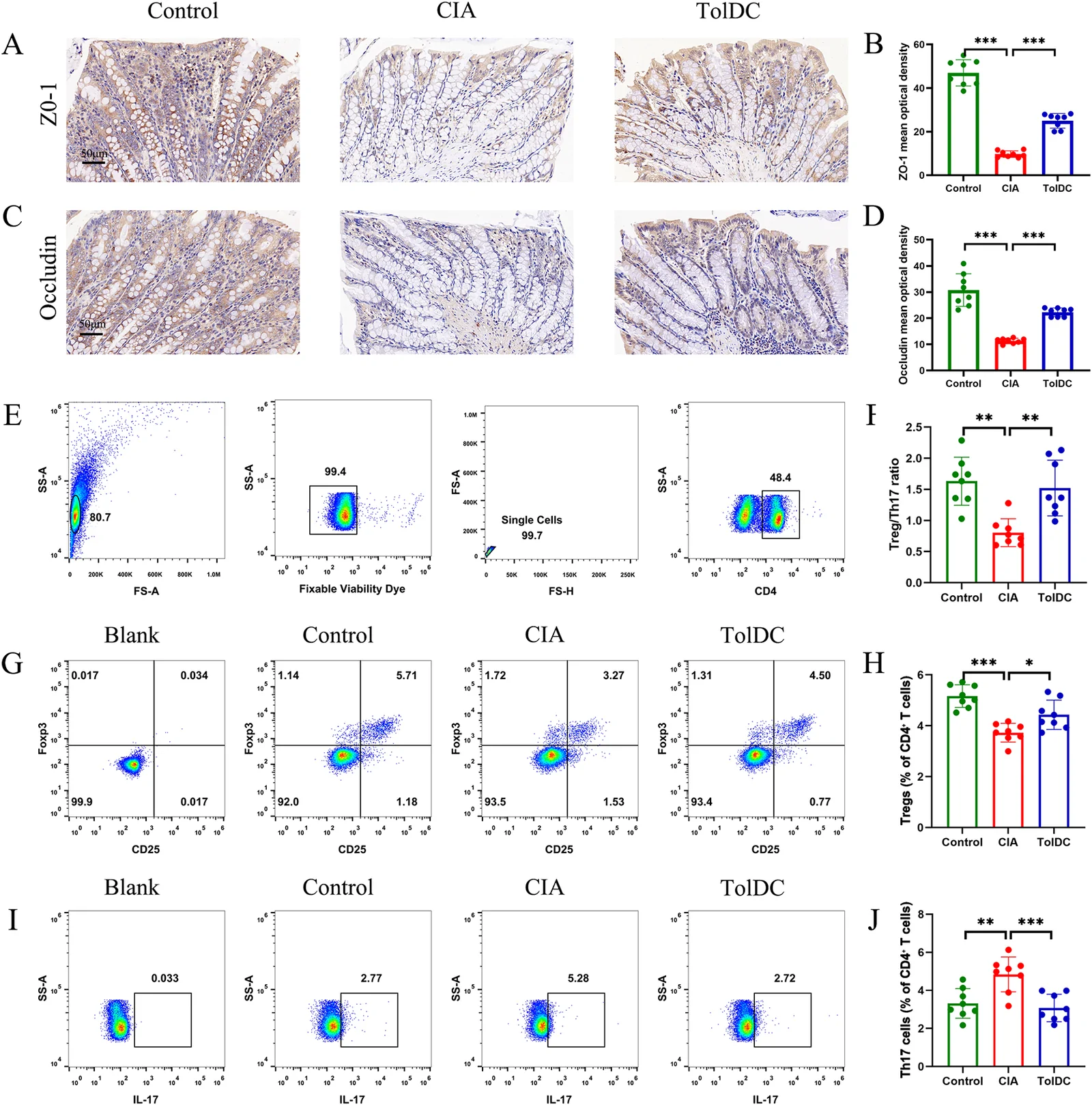
TolDC treatment increases colonic tight-junction protein expression and shifts the MLN Treg/Th17 balance toward a regulatory phenotype. **(A, C)** Representative immunohistochemical staining of ZO-1 and occludin in colon. Scale bars, 50 μm. **(B, D)** Mean optical density of ZO-1 and occludin, calculated as integrated optical density divided by analyzed area. One section per marker per animal was evaluated, and 10 randomly selected, non-overlapping mucosal fields were averaged to one value per animal. **(E)** Flow-cytometry gating strategy: lymphocytes, singlets, viable cells, and CD4^+^ T cells. **(F)** Treg/Th17 ratio. **(G, I)** Representative CD25/Foxp3 and IL-17A plots. Blank denotes an unstained control; fluorescence-minus-one controls were used to establish positive gates and are not shown. **(H)** CD4^+^CD25^+^Foxp3^+^ Tregs and **(J)** CD4^+^IL-17A^+^ Th17 cells, expressed as percentages of viable singlet CD4^+^ T cells. Data are mean ± SEM; n = 8 biologically independent rats per group. Quantitative outcomes were analyzed by one-way ANOVA with Tukey’s multiple-comparisons test. *P < 0.05, **P < 0.01, ***P < 0.001. CIA, collagen-induced arthritis; FSC-A, forward-scatter area; FSC-H, forward-scatter height; MLN, mesenteric lymph node; SSC-A, side-scatter area; Th17, T helper 17 cell; TolDC, tolerogenic dendritic cell; Treg, regulatory T cell.

### 3.6 TolDC treatment alters fecal microbial diversity and community structure

Genus-level core curves showed that the number of genera shared across animals declined as more samples were included, with the CIA group retaining the most and the TolDC group the fewest shared genera (**Figure 6A**). The UpSet plot identified 143 genera common to all three groups, together with pairwise-shared and group-specific genera (**Figure 6B**). CIA rats had a higher Shannon index than Control rats, whereas TolDC treatment reduced Shannon diversity relative to CIA. Chao1 richness did not differ between Control and CIA but was lower in TolDC-treated rats than in CIA rats (**Figures 6C, D**). These changes describe treatment-associated community restructuring and should not be interpreted as inherently beneficial or detrimental.

**Figure 6:**
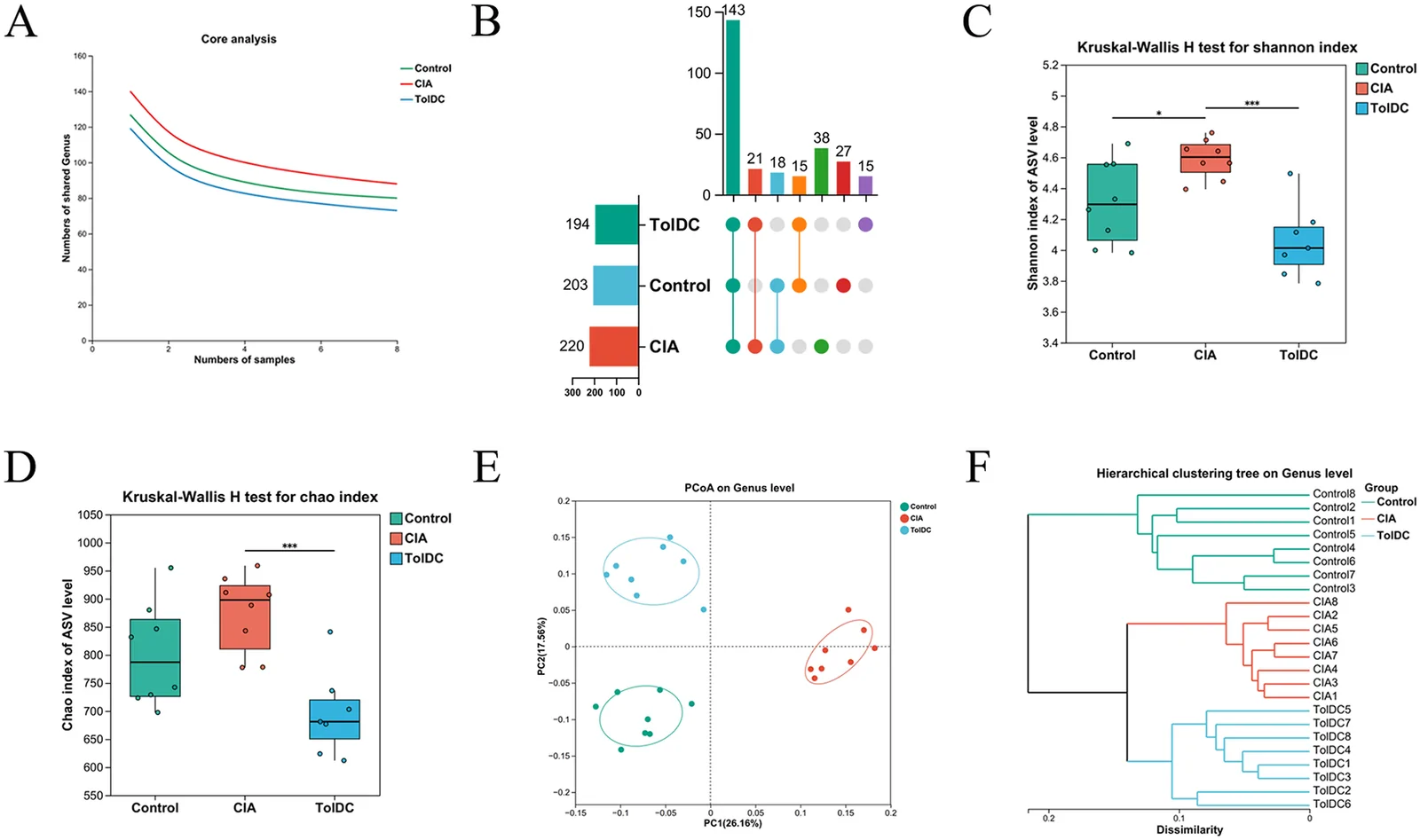
TolDC treatment alters fecal microbial diversity and community structure. **(A)** Core genus curves showing the number of genera shared as increasing numbers of samples were included. **(B)** UpSet plot of shared and group-specific genera; 143 genera were common to the Control, CIA, and TolDC groups. **(C)** Shannon diversity index. **(D)** Chao1 richness index. **(E)** Bray–Curtis principal coordinates analysis (PCoA); PC1 and PC2 explained 26.16% and 17.56% of the variation, respectively. Overall group differences were evaluated by ANOSIM with 999 permutations (P = 0.001). **(F)** Genus-level hierarchical clustering. Analyses used eight biologically independent rats per group (n = 8). Box-and-whisker plots in **(C)** and **(D)** show the median and interquartile range, with whiskers extending to the most extreme values within 1.5 times the interquartile range. Alpha-diversity indices were analyzed by Kruskal–Wallis test with Dunn’s multiple-comparisons test. *P < 0.05; ***P < 0.001. CIA, collagen-induced arthritis; PCoA, principal coordinates analysis; TolDC, tolerogenic dendritic cell.

Bray–Curtis PCoA showed clear separation among Control, CIA, and TolDC samples along PC1 (26.16% of the variation) and PC2 (17.56%), with apparent within-group clustering. ANOSIM confirmed a significant overall difference in community structure among groups (999 permutations, P = 0.001). Genus-level hierarchical clustering further revealed group-associated clustering patterns (**Figures 6E, F**).

### 3.7 TolDC treatment alters phylum- and genus-level microbial composition

Firmicutes predominated across groups, followed by Actinobacteriota, Patescibacteria, and Bacteroidota (**Figure 7A**). After false-discovery-rate correction, phylum-level differences were detected for Firmicutes, Patescibacteria, Bacteroidota, Proteobacteria, and Spirochaetota (**Figure 7B**). Relative-abundance profiles showed lower Firmicutes and higher Patescibacteria and Bacteroidota in CIA than Control rats, whereas the TolDC group shifted in the opposite direction. The Firmicutes-to-Bacteroidota ratio did not differ between Control and CIA but was higher in TolDC-treated rats than in CIA rats (P < 0.001; **Figure 7C**).

**Figure 7:**
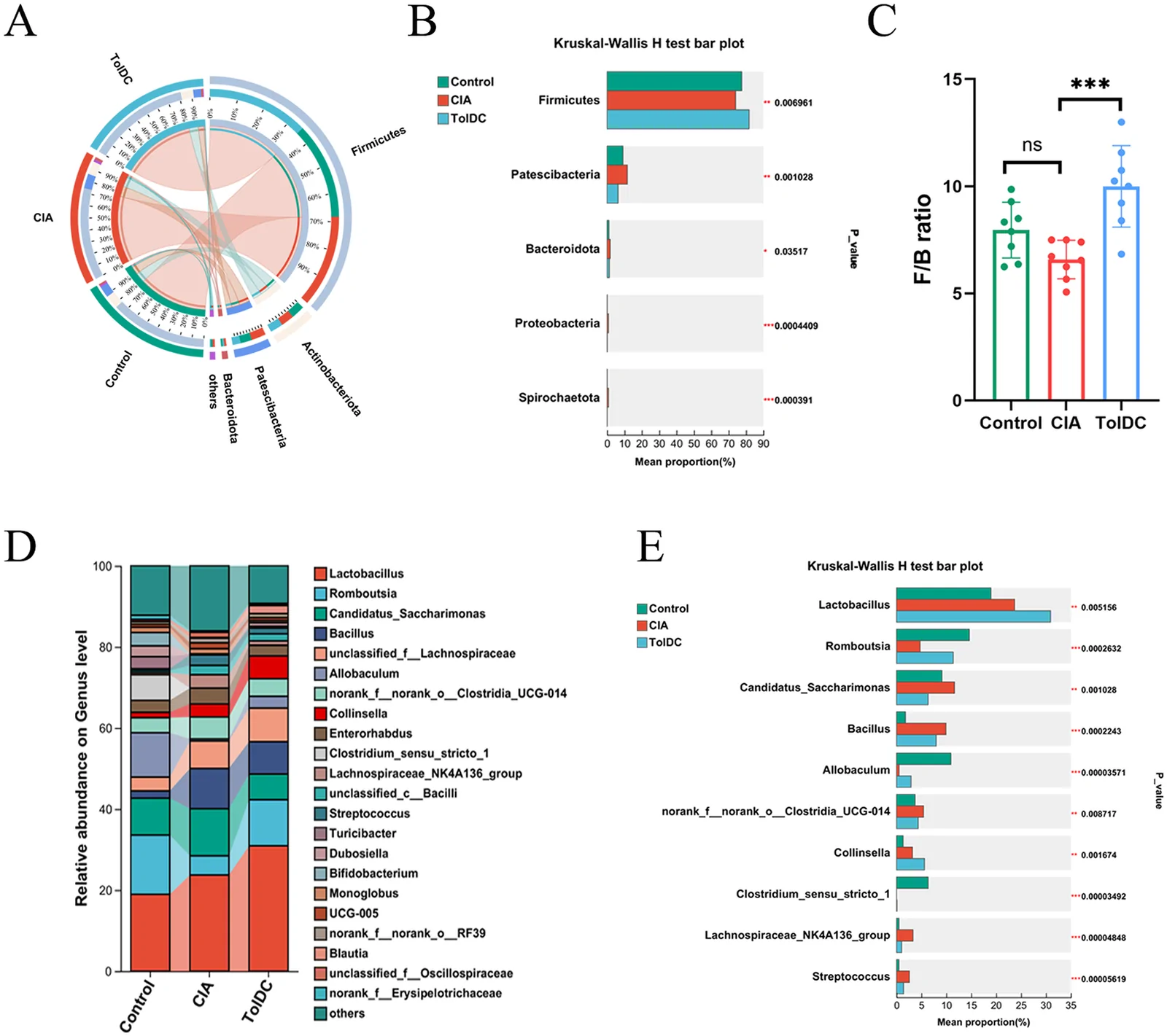
TolDC treatment alters phylum- and genus-level fecal microbial composition. **(A)** Circos plot of predominant bacterial phyla across groups. **(B)** Relative abundances of phyla with significant overall group differences. **(C)** Firmicutes-to-Bacteroidota ratio. **(D)** Genus-level relative-abundance profiles. **(E)** Relative abundances of genera with significant overall group differences. In **(B)** and **(E)**, overall taxon-level differences were assessed using Kruskal–Wallis tests followed by false-discovery-rate (FDR) correction; q < 0.05 was considered significant. The P values displayed in the plots are the Kruskal–Wallis omnibus P values, whereas FDR-adjusted q values were used to determine overall significance. Taxa with significant overall differences were further evaluated using Tukey–Kramer *post-hoc* tests for pairwise group comparisons. Data in **(C)** are mean ± SEM and were analyzed by one-way ANOVA with Tukey’s multiple-comparisons test. Analyses used eight biologically independent rats per group (n = 8). ns, P > 0.05; ***P < 0.001. CIA, collagen-induced arthritis; FDR, false discovery rate; TolDC, tolerogenic dendritic cell.

At the genus level, false-discovery-rate-adjusted overall group differences were identified for Lactobacillus, Romboutsia, Candidatus Saccharimonas, Bacillus, Allobaculum, an unclassified Clostridia UCG-014 taxon, Collinsella, Clostridium sensu stricto 1, the Lachnospiraceae NK4A136 group, and Streptococcus (**Figures 7D, E**). Tukey–Kramer *post-hoc* comparisons showed that, relative to CIA rats, TolDC-treated rats had lower relative abundances of Candidatus Saccharimonas, the unclassified Clostridia UCG-014 taxon, and Streptococcus, together with higher relative abundances of Lactobacillus, Romboutsia, and Allobaculum. The remaining taxa showed significant overall group differences but were not interpreted as specific CIA-versus-TolDC changes unless supported by *post-hoc* pairwise comparisons. These findings demonstrate broad taxonomic remodeling but do not establish strain-level function or metabolite production.

### 3.8 TolDC treatment alters predicted gut microbial SCFA-related metabolic functions and circulating SCFA profiles

To explore possible changes in gut microbial SCFA-related metabolic potential, PICRUSt2 predictions were assessed for selected MetaCyc pathways and KO functional groups in the Control, CIA, and TolDC groups (**Figures 8A, B**). At the pathway level, the acetate-associated PWY-5100 and propionate-associated PWY-7013 showed higher predicted abundances in TolDC-treated rats than in the indicated comparator groups, whereas the mixed-acid fermentation-associated FERMENTATION-PWY was lower in TolDC than CIA; P108-PWY did not show significant differences. Notably, the butyrate-associated pathways PWY-5676 and CENTFERM-PWY were more abundant in TolDC-treated rats than in both the Control and CIA groups (**Figure 8A**). In the KO analysis, K13922, which is associated with a propanediol-related propionate pathway, was higher in TolDC than CIA. Several butyrate-associated functions (K00626, K00074, and K00634) were also higher in TolDC than CIA, while the selected acetate-associated KOs (K00625 and K00925) did not show significant differences (**Figure 8B**).

**Figure 8:**
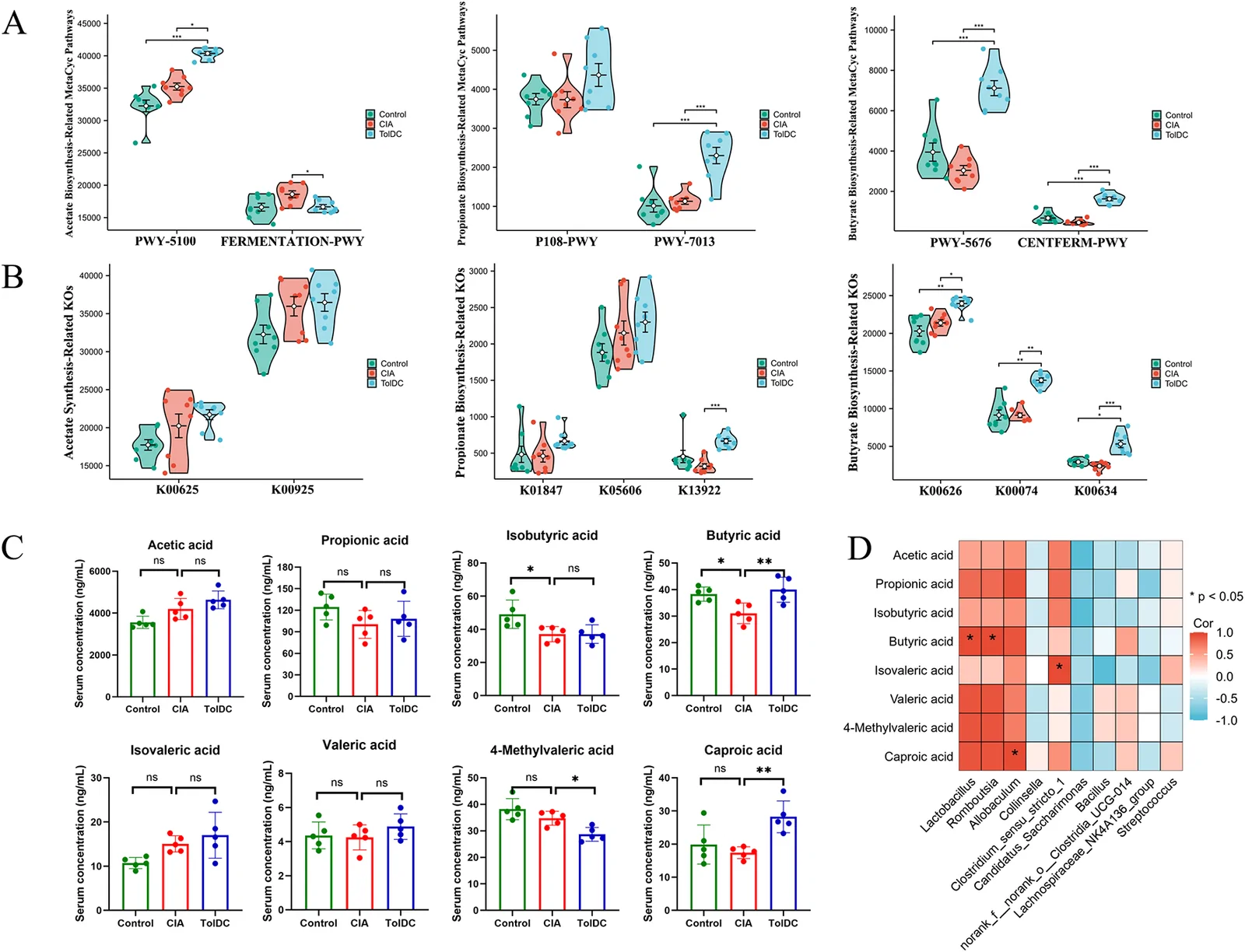
TolDC treatment alters predicted gut microbial SCFA-related metabolic functions and circulating SCFA profiles. **(A)** PICRUSt2-predicted abundances of MetaCyc pathways associated with acetate (PWY-5100 and FERMENTATION-PWY), propionate (P108-PWY and PWY-7013), and butyrate (PWY-5676 and CENTFERM-PWY) -related metabolism in the Control, CIA, and TolDC groups. **(B)** PICRUSt2-predicted abundances of selected KEGG Orthology (KO) functional genes associated with acetate (K00625 and K00925), propionate (K01847, K05606, and K13922), and butyrate (K00626, K00074, and K00634) -related metabolism. For panels A and B, differences among groups were assessed using the Kruskal–Wallis test followed by Dunn’s *post hoc* multiple-comparisons test. **(C)** Serum concentrations of acetate, propionate, isobutyrate, butyrate, isovalerate, valerate, 4-methylvalerate, and caproate measured by targeted metabolomics. Five non-hemolyzed serum samples were analyzed per group (n = 5); hemolyzed specimens were excluded from targeted SCFA quantification. Serum SCFA concentrations were analyzed using one-way ANOVA followed by Tukey’s multiple-comparisons test. **(D)** Spearman correlation heatmap illustrating associations between circulating SCFA concentrations and differentially abundant bacterial genera based on paired microbiome and serum metabolomic data from 15 rats (five per group). Red indicates positive correlations, whereas blue indicates negative correlations. Correlation P values were adjusted for multiple testing, and asterisks indicate associations that remained significant after adjustment. For panels A–C, ns, P > 0.05; *P < 0.05; **P < 0.01; ***P < 0.001. For panel D, * indicates an adjusted P < 0.05. CIA, collagen-induced arthritis; KO, KEGG Orthology; PICRUSt2, Phylogenetic Investigation of Communities by Reconstruction of Unobserved States 2; SCFA, short-chain fatty acid; TolDC, tolerogenic dendritic cell.

Targeted serum SCFA profiling was performed in five non-hemolyzed serum samples per group. Serum SCFA profiling showed lower isobutyrate and butyrate in CIA rats than in Control rats (both P < 0.05; **Figure 8C**). TolDC treatment increased butyrate and caproate relative to CIA (both P < 0.01; **Figure 8C**) and decreased 4-methylvalerate (P < 0.05); acetate, propionate, and isovalerate did not differ in the indicated comparisons. Butyrate therefore showed both a disease-associated reduction and a treatment-associated increase. Spearman analysis of paired microbiome and serum metabolomic data from 15 rats identified positive associations of butyrate with Lactobacillus and Romboutsia, isovalerate with Clostridium sensu stricto 1, and caproate with Allobaculum that remained significant after multiple-testing correction (**Figure 8D**). These adjusted correlations were interpreted as exploratory and do not establish direct metabolite production by the correlated genera or a causal relationship.

### 3.9 Sodium butyrate partially recapitulates the TolDC-associated protective phenotype

Because butyrate was reduced in CIA and increased after TolDC treatment, we tested whether exogenous sodium butyrate could reproduce selected therapeutic effects. Rats in the Butyrate group received sodium butyrate from day 21 to day 49 (**Figure 9A**). On day 49, right hind-paw swelling was less pronounced than in the CIA group (**Figure 9B**), and hind-ankle width and arthritis scores were lower on days 35, 42, and 49 (**Figures 9C, D**). Butyrate-treated joints also showed less synovial hyperplasia and inflammatory-cell infiltration and greater structural preservation (**Figure 9E**). Histopathological scores were lower than in CIA but remained higher than in Control rats (both P < 0.001; **Figure 9F**). Thus, sodium butyrate partially recapitulated the TolDC-associated protective phenotype, although the two interventions were not compared directly within the same cohort.

**Figure 9:**
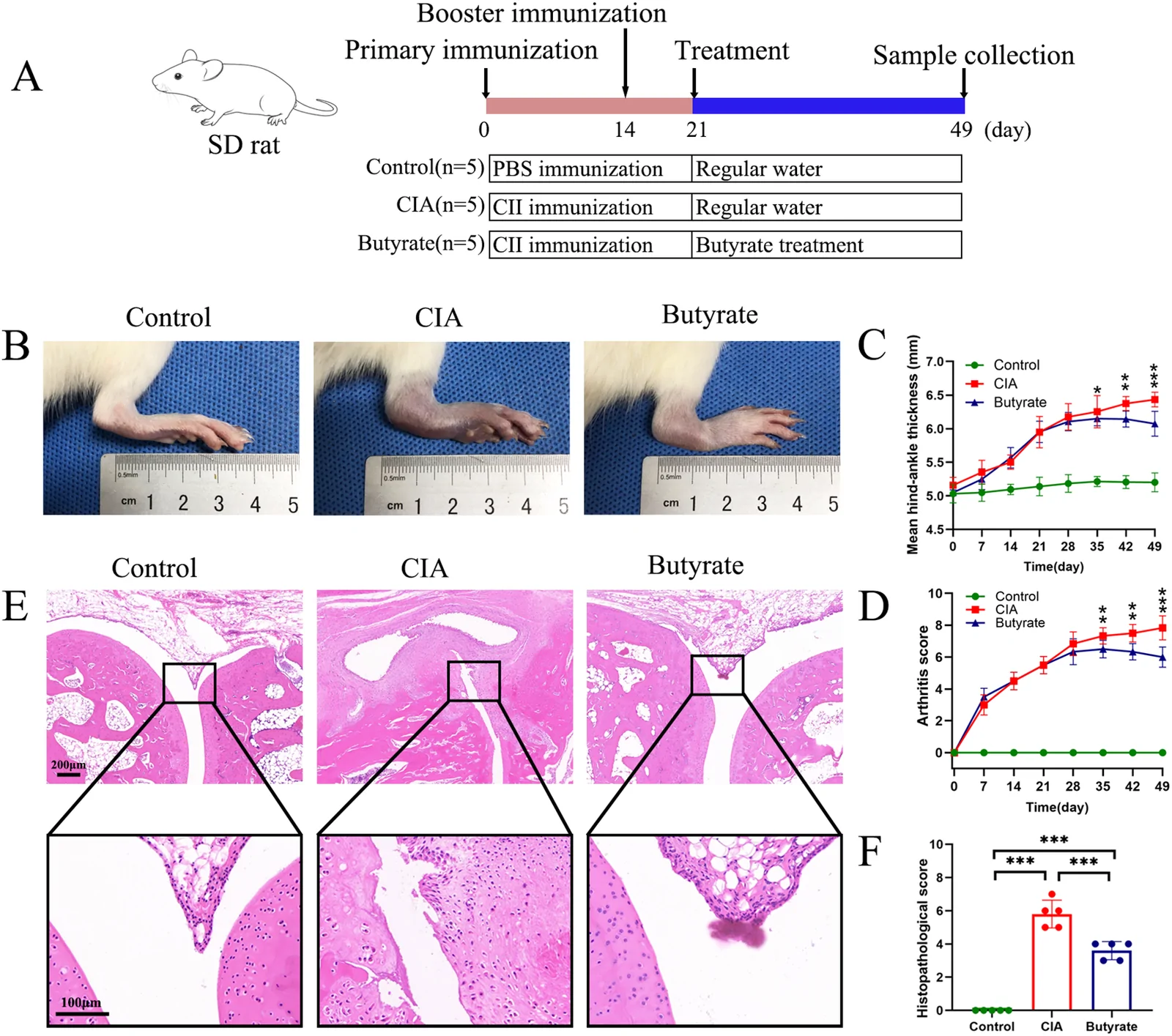
Sodium butyrate partially recapitulates the TolDC-associated protective phenotype in CIA rats. **(A)** Experimental timeline. Control rats received PBS at the corresponding immunization time points; CIA and Butyrate groups received CII on days 0 and 14. From day 21 to day 49, rats received regular drinking water or water containing 150 mM sodium butyrate. Samples were collected on day 49. **(B)** Representative right hind paws on day 49. **(C)** Mean width of both hind ankles. **(D)** Total arthritis scores (maximum, 16). **(E)** Representative hematoxylin and eosin-stained ankle joints. Scale bars, 200 μm in the upper panels and 100 μm in the enlarged lower panels. **(F)** Composite histopathological scores (range, 0–6). Data are mean ± SEM; n = 5 biologically independent rats per group. Longitudinal outcomes were analyzed by two-way repeated-measures ANOVA with Šídák’s comparisons. Asterisks in **(C)** and **(D)** denote CIA-versus-Butyrate comparisons at the corresponding time points. Histopathological scores were analyzed by one-way ANOVA with Tukey’s test. *P < 0.05, **P < 0.01, ***P < 0.001. CIA, collagen-induced arthritis; CII, type II collagen; TolDC, tolerogenic dendritic cell.

## 4 Discussion

Accumulating evidence links rheumatoid arthritis (RA) to gut microbial dysbiosis, altered microbial metabolism, and impaired intestinal barrier integrity (27, 28). Microbiota-derived short-chain fatty acids (SCFAs) can reach the systemic circulation and exert immunoregulatory and osteoimmune effects, thereby providing a potential metabolic link between the intestine and the joint (29, 30).

The present study extends the mechanism of antigen-loaded tolerogenic dendritic cell (TolDC) therapy beyond direct systemic immunoregulation to the gut–joint axis. Collagen-loaded TolDCs attenuated paw swelling, clinical arthritis, and ankle-joint histopathology in rats with collagen-induced arthritis (CIA). This protection was accompanied by increased colonic ZO-1 and occludin expression, a shift in the mesenteric lymph node (MLN) Treg/Th17 balance toward a regulatory profile, remodeling of fecal microbial communities, and an increase in serum butyrate. Importantly, under concurrent antibiotic treatment, TolDC-associated improvements in arthritis outcomes did not reach statistical significance, whereas fecal microbiota transplantation (FMT) from TolDC-treated donors partially transferred protection to antibiotic-pretreated recipients. Sodium butyrate supplementation also reproduced part of the clinical and histopathological improvement. Together, these findings support a model in which mucosal immunity, microbial ecology, and microbial metabolism form an interacting component of TolDC-mediated disease control rather than merely changing in parallel with treatment response.

TolDCs are designed to restore antigen-specific immune tolerance while avoiding the broad immunosuppression associated with many conventional anti-inflammatory treatments. Their therapeutic activity is commonly associated with an immature or semimature phenotype, reduced costimulatory signaling, impaired effector T-cell activation, and promotion of regulatory immune responses (31, 32). The NF-κB decoy oligodeoxynucleotide-induced cells generated in the present study retained high OX-62 expression but showed lower frequencies of CD80- and CD86-expressing cells, consistent with an immature DC phenotype. Their ability to reduce CIA severity agrees with previous studies showing that antigen-loaded TolDCs can suppress autoimmune joint inflammation and promote immune tolerance in experimental arthritis (26, 33). The present findings extend this framework by indicating that intravenously administered TolDCs are associated not only with systemic immune regulation but also with changes in the intestinal immune and microbial environment.

The combined antibiotic-treatment and fecal microbiota transplantation (FMT) experiments provide complementary, although not definitive, evidence supporting the contribution of the gut microbiota to TolDC-mediated therapeutic effects. TolDC treatment alleviated arthritis in CIA rats without antibiotic exposure. Under antibiotic treatment, the TolDC + ABX group exhibited numerically lower measures of disease severity than the ABX group, although these differences did not reach statistical significance. Importantly, the absence of a statistically significant therapeutic effect under antibiotic treatment does not indicate a complete loss of TolDC activity, nor does it, by itself, establish a significant interaction between antibiotic exposure and TolDC treatment. Nevertheless, together with the FMT findings, these results support an important contributory role of the gut microbiota in enabling TolDCs to exert their full therapeutic effects.

The FMT experiment provided complementary evidence in the opposite direction. Microbiota transferred from TolDC-treated donors reduced paw swelling, arthritis scores, and joint histopathology in antibiotic-pretreated CIA recipients, whereas microbiota from untreated CIA donors did not produce a comparable improvement. The transferred protection was partial rather than complete, which is biologically plausible because TolDCs may also act through microbiota-independent mechanisms, including direct modulation of antigen presentation and systemic T-cell responses. Taken together, the absence of a statistically detectable TolDC advantage during antibiotic exposure and the functional transfer evidence provided by FMT from TolDC-treated donors move the evidence beyond compositional associations detected by 16S rRNA sequencing and support a microbiota-dependent component of TolDC therapy. Similar combinations of antibiotic-mediated microbiota depletion and FMT into antibiotic-pretreated recipients have been used to determine whether treatment- or disease-associated microbial changes contribute functionally to inflammatory arthritis phenotypes (34, 35).

Nevertheless, these experiments do not establish the gut microbiota as the sole determinant of TolDC efficacy. Broad-spectrum antibiotics can affect host physiology through both microbiota-dependent and microbiota-independent mechanisms, including direct effects on intestinal epithelial cells and mitochondrial or metabolic processes (36). Furthermore, FMT transfers a complex fecal ecosystem comprising viable microorganisms, bacteriophages, microbial metabolites and products, and other non-bacterial fecal components (37, 38). Therefore, the results support a possible contributory role for the gut microbiota in TolDC-associated protection, but they do not identify the specific organisms or transferable components responsible for the phenotype. The involvement of the microbiota is consistent with the growing recognition of the gut–joint axis in RA. Intestinal dysbiosis, impaired epithelial integrity, altered microbial metabolites, and abnormal mucosal immune activation have all been associated with RA development or disease activity (39). Increased intestinal permeability may increase systemic exposure to microbial products, thereby promoting innate immune activation and inflammatory T-cell responses at distant sites, including the synovium (40, 41). Conversely, host epithelial and immune responses can reshape the intestinal niche by altering barrier function, nutrient availability, antimicrobial activity, and immune selection of microbial communities (42, 43). The present results therefore suggest that TolDCs may influence the gut–joint axis through two interconnected processes: direct modulation of host immune regulation and indirect remodeling of the mucosal environment that supports the intestinal microbial community.

The changes observed in the colon and MLNs provide a plausible immunological bridge between TolDC administration and microbial remodeling. CIA was associated with reduced colonic ZO-1 and occludin expression, whereas both tight-junction proteins increased after TolDC treatment. Although immunohistochemical detection of tight-junction proteins does not directly measure intestinal permeability, these findings are consistent with improvement of a barrier-associated epithelial phenotype. Disruption of epithelial tight junctions may increase intestinal permeability and host exposure to microbial products, thereby amplifying systemic inflammatory responses, whereas preservation of epithelial integrity may help restrict this inflammatory input (44, 45).

TolDC treatment also increased the frequency of CD4^+^CD25^+^Foxp3^+^ Tregs, reduced CD4^+^IL-17A^+^ Th17 cells, and increased the Treg/Th17 ratio in MLNs. The MLNs are key gut-draining immune sites where intestinal antigens and microbiota-derived signals are presented and integrated to shape adaptive immune responses and mucosal tolerance (46, 47). Tregs help maintain mucosal tolerance and restrain excessive immune activation, whereas Th17 cells promote mucosal inflammation and contribute to synovitis, RANKL-dependent osteoclastogenesis, and joint destruction in RA (48, 49). A TolDC-associated shift toward regulatory immunity in the MLNs could therefore reduce both local mucosal inflammation and systemic arthritogenic immune activation. At the same time, improved epithelial integrity and reduced mucosal inflammatory pressure may favor more stable host–microbiota interactions. These observations support a bidirectional immune–microbial relationship rather than a strictly linear pathway in which immune regulation simply precedes or follows microbial change.

Microbiota profiling further showed that TolDC treatment altered both alpha diversity and overall community structure. CIA rats exhibited higher Shannon diversity than Control rats, whereas TolDC treatment reduced both Shannon diversity and Chao1 richness relative to CIA. This pattern reinforces that greater microbial diversity is not inherently beneficial. Diversity indices describe ecological characteristics of a community but do not identify the organisms or functions responsible for a disease phenotype (50). The biological significance of the TolDC-associated microbial community should therefore be evaluated together with its taxonomic composition, metabolic profile, and functional transferability.

At the phylum level, CIA rats exhibited a lower relative abundance of Firmicutes and higher relative abundances of Bacteroidota and Patescibacteria than Control rats, whereas TolDC treatment shifted these taxa in the opposite direction. Although the Firmicutes-to-Bacteroidota (F/B) ratio did not differ significantly between the Control and CIA groups, it was significantly higher in the TolDC group than in the CIA group. This increase therefore does not represent a simple restoration of a pre-existing CIA-associated abnormality. Rather, it reflects a distinct treatment-associated reorganization of the gut microbial community.

The F/B ratio provides a broad ecological measure of the relative balance between two dominant bacterial phyla and may therefore serve as a useful descriptive indicator of community-level remodeling (51, 52). In the present study, its increase, together with the partial recovery of Firmicutes and reduction of Bacteroidota, suggests that TolDC treatment shifted the intestinal ecosystem toward a microbial configuration associated with CIA attenuation. Nevertheless, because the biological significance of the F/B ratio is context dependent, it should be interpreted as supportive evidence of microbial community restructuring rather than as a stand-alone marker of gut health or a direct mediator of TolDC efficacy (52). Its biological relevance should therefore be considered in conjunction with genus- or species-level changes, microbial metabolic profiles, and host immune responses (53).

At the genus level, TolDC treatment lowered the relative abundances of several taxa enriched in CIA, including *Candidatus Saccharimonas*, the unclassified *Clostridia UCG-014* taxon, and *Streptococcus*. These taxa have previously been reported to be altered in inflammatory or immune-related conditions (54–56). In parallel, TolDC treatment increased *Lactobacillus*, *Romboutsia*, and *Allobaculum*, genera that have been linked, in species- and model-dependent studies, to carbohydrate utilization, SCFA-related metabolic states, or intestinal immune regulation (57–59). These treatment-associated shifts are consistent with the possibility that TolDCs promote the reorganization of microbial networks associated with immune homeostasis and microbial metabolite availability. One possible explanation is that adoptively transferred TolDCs modulate gut-associated immune circuits, including dendritic-cell–T-cell interactions in the mesenteric lymph nodes, thereby altering the intestinal mucosal environment and indirectly reshaping microbial ecology (17, 60).

The SCFA findings provide a plausible, exploratory metabolic link between microbial remodeling and host outcomes. PICRUSt2 predicted higher abundances of selected butyrate-related MetaCyc pathways (PWY-5676 and CENTFERM-PWY) and KOs (K00626, K00074, and K00634) after TolDC treatment, accompanying increased circulating butyrate. However, these predictions derive from 16S-based inference and do not demonstrate *in vivo* gene expression, enzyme activity, or actual microbial SCFA production. Serum concentrations are also affected by epithelial uptake, absorption, hepatic metabolism, tissue use, and systemic clearance. Correlations between serum butyrate and Lactobacillus or Romboutsia after multiple-testing correction likewise do not establish direct production by those genera or a causal relation to arthritis improvement. The concordance of predicted features and serum butyrate therefore supports biological plausibility rather than mechanistic proof.

Butyrate is particularly relevant to RA because it may act at several points along the proposed gut–joint pathway. It is an important energy source for colonic epithelial cells, supports tight-junction integrity, promotes regulatory T-cell differentiation, and suppresses inflammatory signaling through G protein-coupled receptors and inhibition of histone deacetylases (61, 62). Butyrate can also restrain inflammatory cytokine programs and modulate osteoclast differentiation and bone–immune interactions, processes that are directly relevant to cartilage and bone damage in RA (20).

The sodium butyrate intervention strengthens the functional interpretation of the metabolomic findings. Sodium butyrate supplementation reduced paw swelling, arthritis scores, and joint histopathology, thereby partially recapitulating the protective phenotype observed after TolDC administration. Thus, butyrate was not merely a biomarker that changed in parallel with disease severity; increasing butyrate availability was sufficient to improve selected clinical and histopathological outcomes. However, sodium butyrate reproduced only part of the TolDC-associated phenotype, and the two interventions were not directly compared within the same experimental cohort. Moreover, the present study did not include butyrate depletion, receptor blockade, inhibition of butyrate signaling, or an antibiotic-plus-butyrate rescue experiment. The results therefore support butyrate as a functional contributor to arthritis amelioration and a candidate component of TolDC-associated protection, but they do not establish that endogenous butyrate is required for TolDC efficacy or that it is the sole mediator of the therapeutic response.

Overall, the results are compatible with reciprocal immune–microbial–metabolic interactions rather than a demonstrated linear pathway. TolDC-associated changes in regulatory immunity and barrier-associated proteins could influence the mucosal environment, while shifts in microbial functional potential and circulating SCFAs may accompany disease attenuation. The cross-sectional endpoint assessments cannot determine which changes precede or mediate the others. Longitudinal sampling and specific microbial, cellular, and SCFA-targeted interventions would be needed to establish temporal and direct molecular connections.

Several limitations should be acknowledged. First, characterization of the NF-κB decoy ODN-treated TolDCs was primarily phenotypic in the present study. Neither intact intracellular decoy ODN persistence and NF-κB inhibition kinetics nor the ability of TolDCs to retain tolerogenic function after confirmed ODN clearance was assessed. Moreover, CII-reactive T-cell proliferation, cytokine production, antigen-specific Treg/Th17 differentiation, and their functional stability after inflammatory challenge were not directly evaluated; prior mixed lymphocyte reaction, LPS-response, and *in vivo* T-cell findings provide only indirect support for these functions. Second, 16S rRNA sequencing lacks strain-level resolution, and PICRUSt2-based MetaCyc and KO profiles are predictions of microbial functional potential rather than direct measurements of microbial gene expression or SCFA production. Third, targeted serum SCFA profiling included only five non-hemolyzed rats per group (from an initial eight per group), limiting precision and potentially introducing selection bias. Serum SCFAs do not directly measure intestinal production, and corresponding fecal or intestinal-content SCFA profiles were unavailable. Genus–metabolite correlations in 15 paired samples remain exploratory. Fourth, the antibiotic experiment does not exclude antibiotic effects on the host or demonstrate that TolDC action is exclusively microbiota-dependent; pooled-donor FMT also limits donor-level inference. Finally, the temporal sequence linking TolDCs, mucosal immunity, epithelial cells, microbial functions, and metabolites was not resolved, and the necessity of endogenous butyrate for TolDC protection was not tested. Future research should assess decoy kinetics and CII-specific function, perform longitudinal strain- and function-resolved analyses, and combine intestinal and circulating metabolomics with direct microbiota and metabolite perturbations.

In conclusion, NF-κB decoy ODN-treated TolDCs attenuated experimental arthritis in association with changes in mucosal immune balance, gut microbial ecology, predicted butyrate-related functional potential, and circulating butyrate. During antibiotic exposure, numerical improvements were observed but did not reach statistical significance, whereas FMT from TolDC-treated donors partially transferred protection. Exogenous sodium butyrate also improved selected arthritis outcomes. These findings support further study of an interacting TolDC–mucosal immunity–microbiota–butyrate model; its antigen specificity, cellular stability, microbial metabolite production, and direct causal links remain to be established (**Figure 10**).

**Figure 10:**
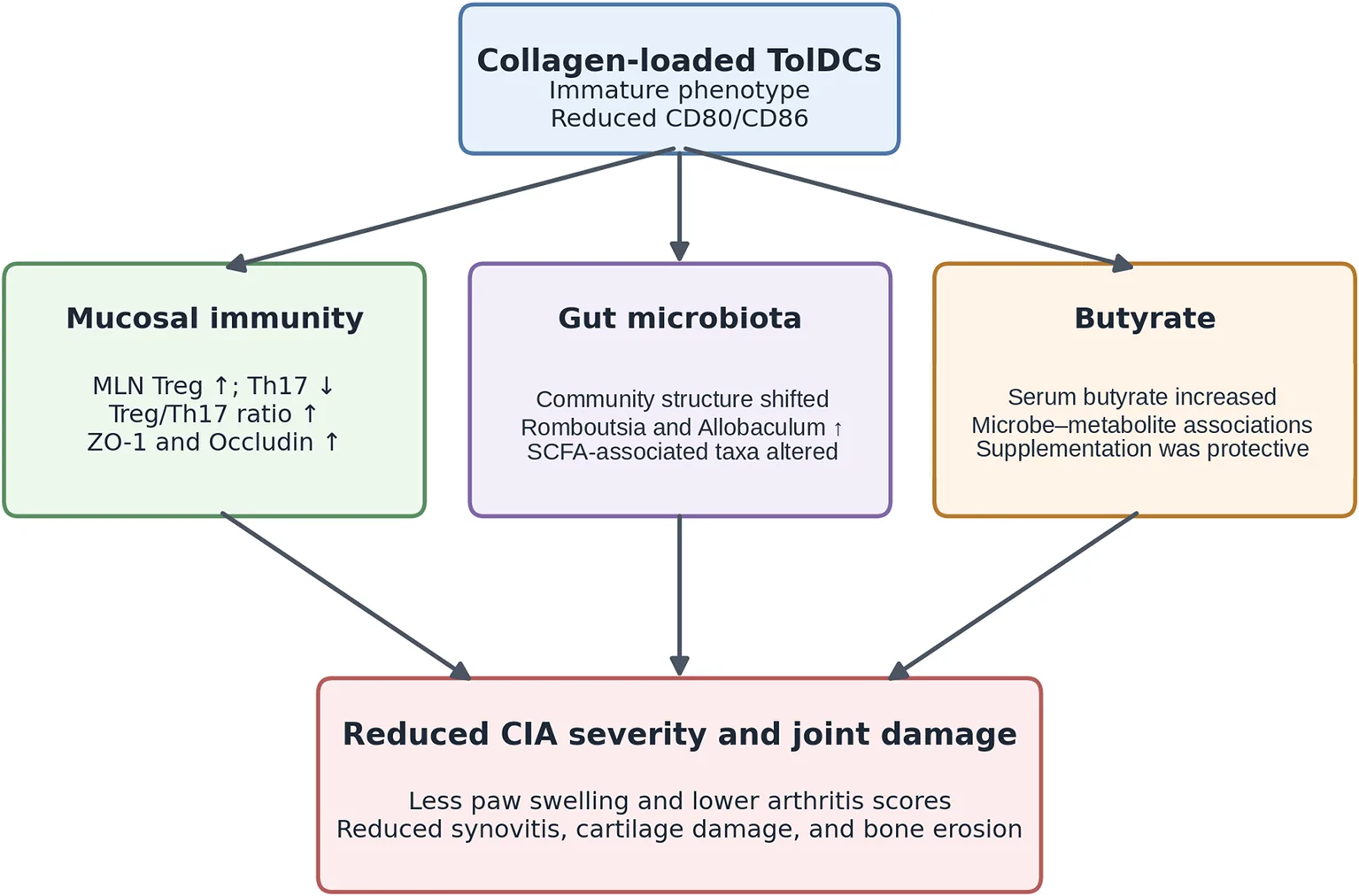
Proposed working model of TolDC-associated protection in CIA. The working model links CII-loaded TolDC administration with a regulatory shift in MLN Treg/Th17 balance, higher colonic ZO-1 and occludin expression, altered gut microbial communities and predicted SCFA-related functions, and increased circulating butyrate. These associated changes may jointly relate to lower synovial inflammation and joint pathology; the arrows illustrate a proposed rather than experimentally established causal sequence. Antibiotic-mediated microbiota depletion, FMT, and sodium butyrate supplementation provide functional support for the model, although the temporal order and all intermediate molecular steps were not directly tested. CIA, collagen-induced arthritis; CII, type II collagen; FMT, fecal microbiota transplantation; MLN, mesenteric lymph node; TolDC, tolerogenic dendritic cell.

## Data Availability

The 16S rRNA gene-sequencing data generated in this study are available in the NCBI Sequence Read Archive under BioProject accession PRJNA1110009. The source data underlying the clinical, histopathological, flow cytometric, immunohistochemical, and targeted metabolomic analyses will be made available by the authors upon reasonable request, without undue reservation.
